# Supplementary material for: A Study of the Chemical Composition and Biological Activity of Michelia macclurei Dandy Heartwood: New Sources of Natural Antioxidants, Enzyme Inhibitors and Bacterial Inhibitors
Source: Int J Mol Sci. 2023 Apr 28;24(9):7972. doi: 10.3390/ijms24097972 (PMC10177984; doi:10.3390/ijms24097972)

Supplementary Figure S1. MS/MS spectrum and Proposed fragmentation pathways of 35 isoquinoline alkaloids

Compounds 2: (S)-Scoulerine

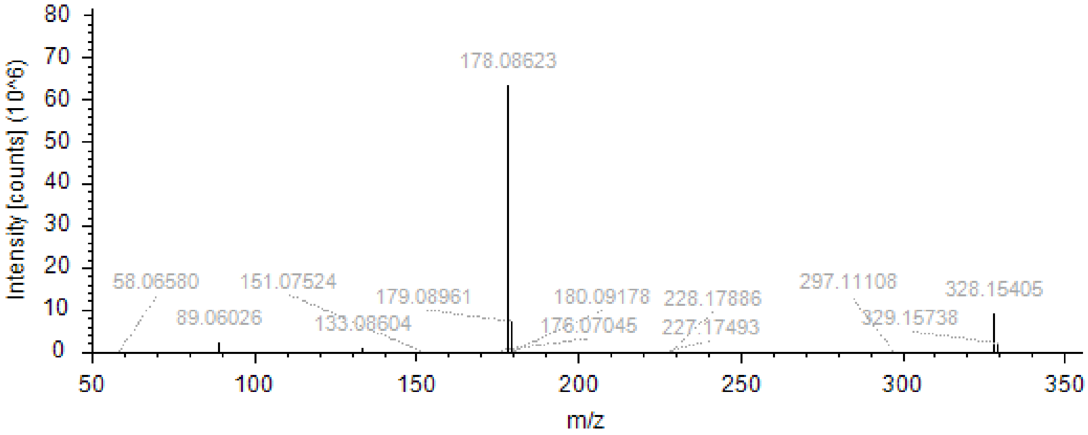

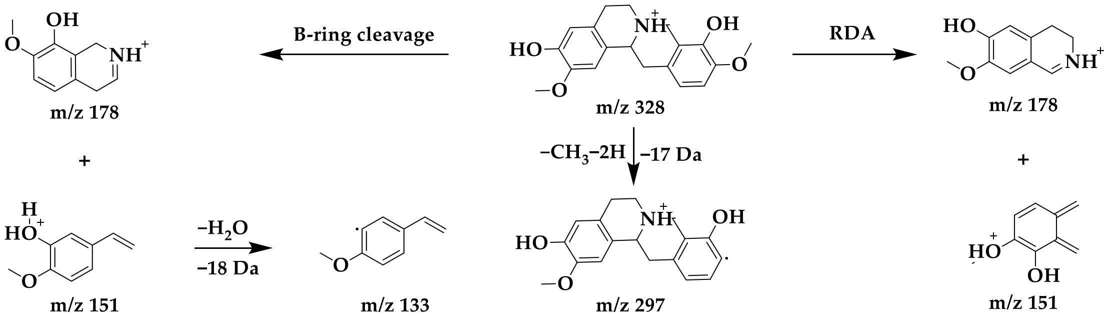

Compounds 3: Magnoflorine

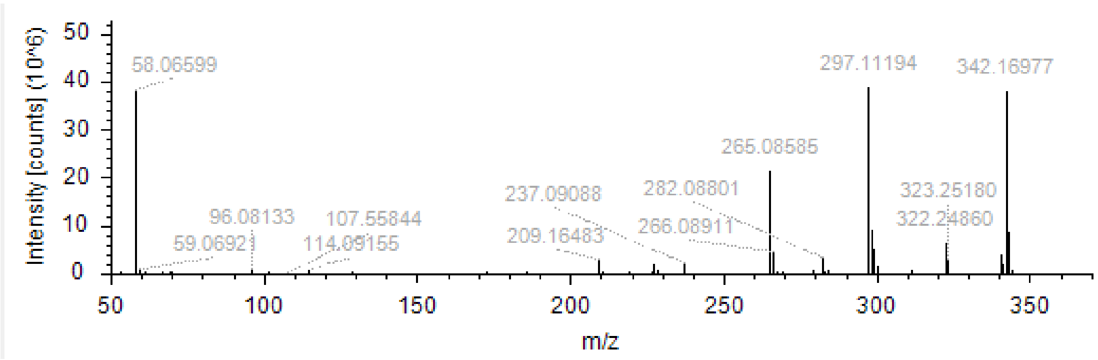

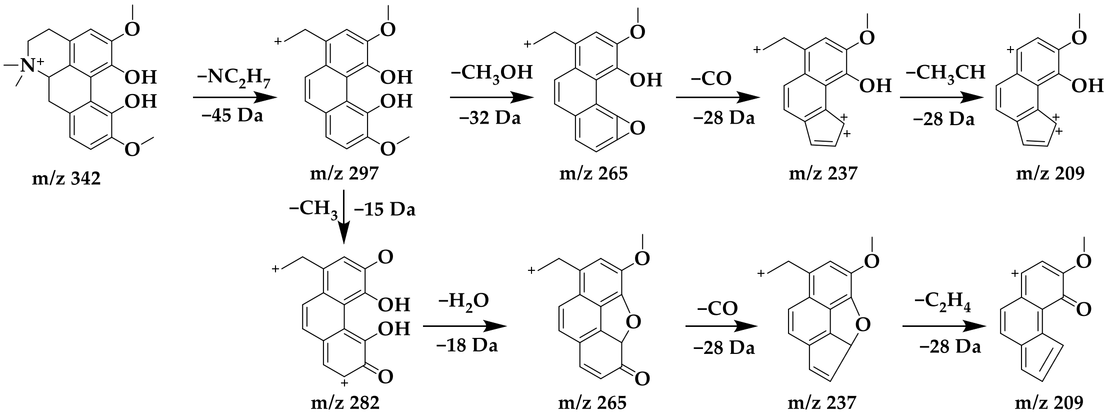

Compounds 4: (S)-Reticuline

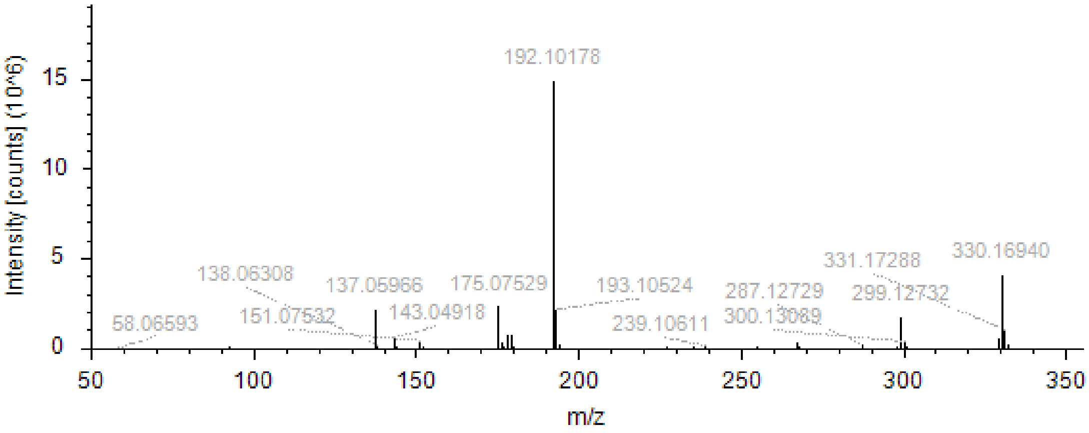

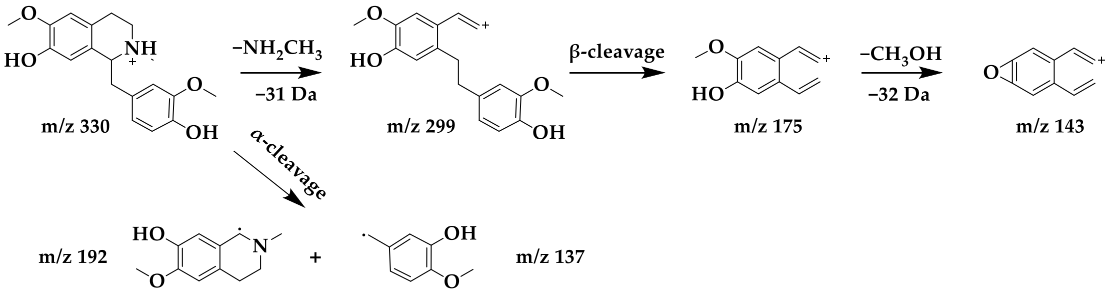

Compounds 6: Laurifoline

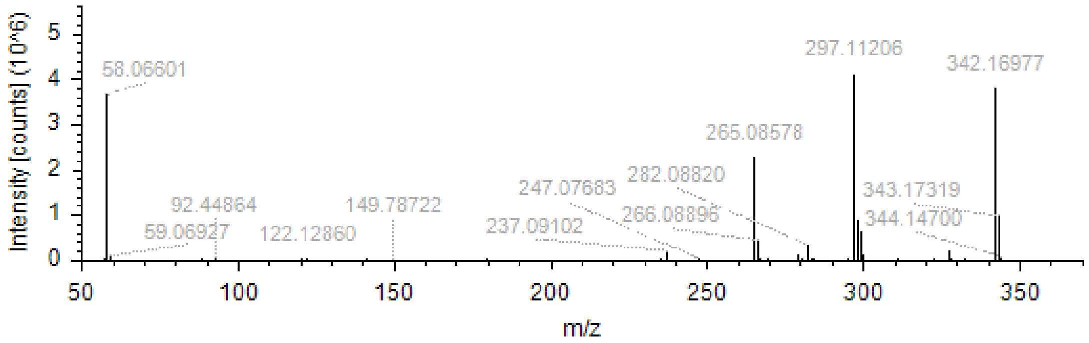

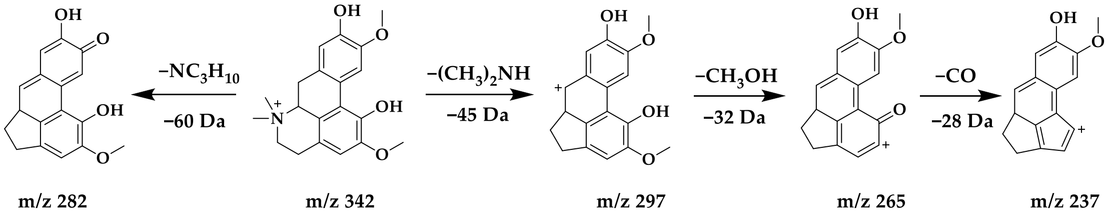

Compounds 7: Corytuberine

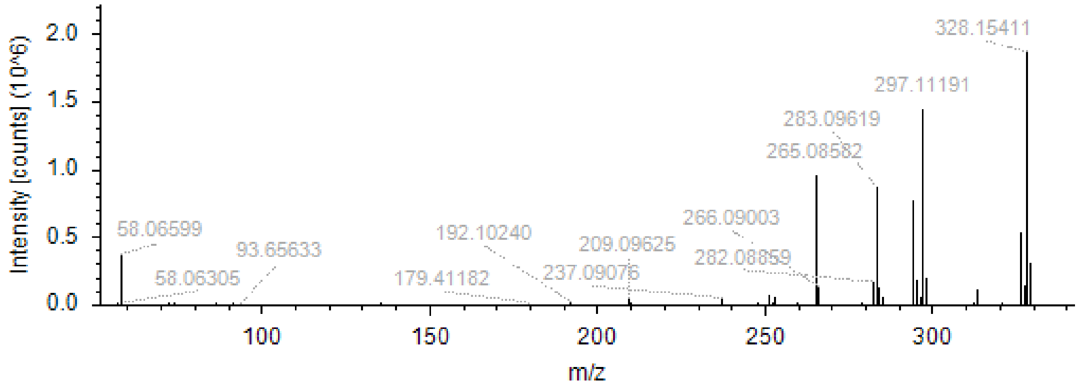

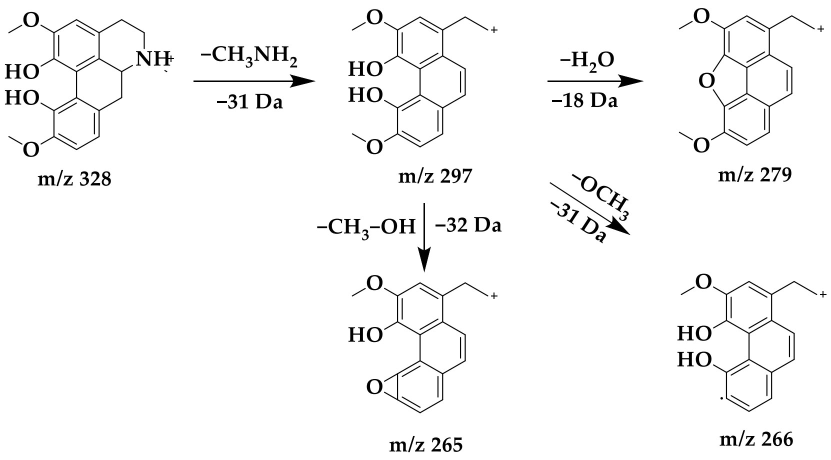

Compounds 8: N,N-Dimethylglaucine

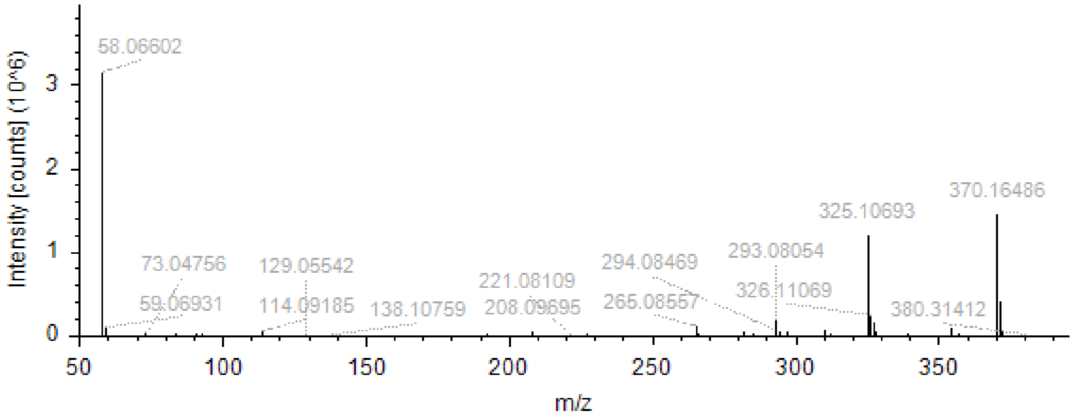

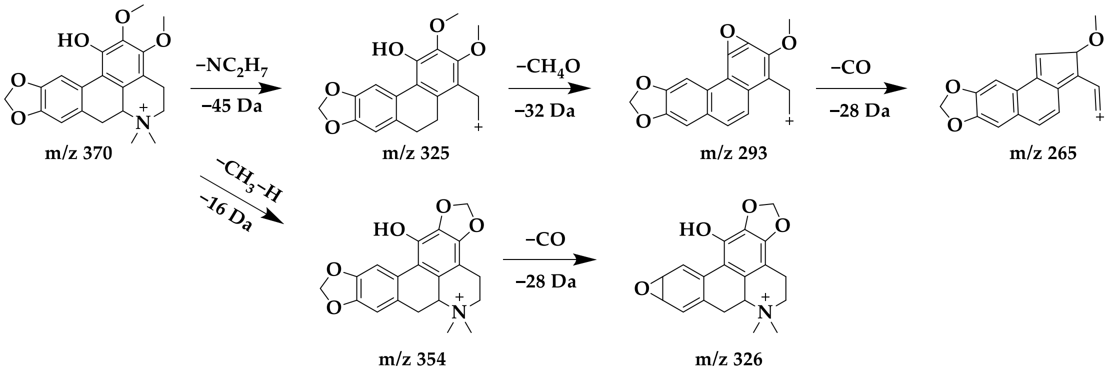

Compounds 9: Protopine

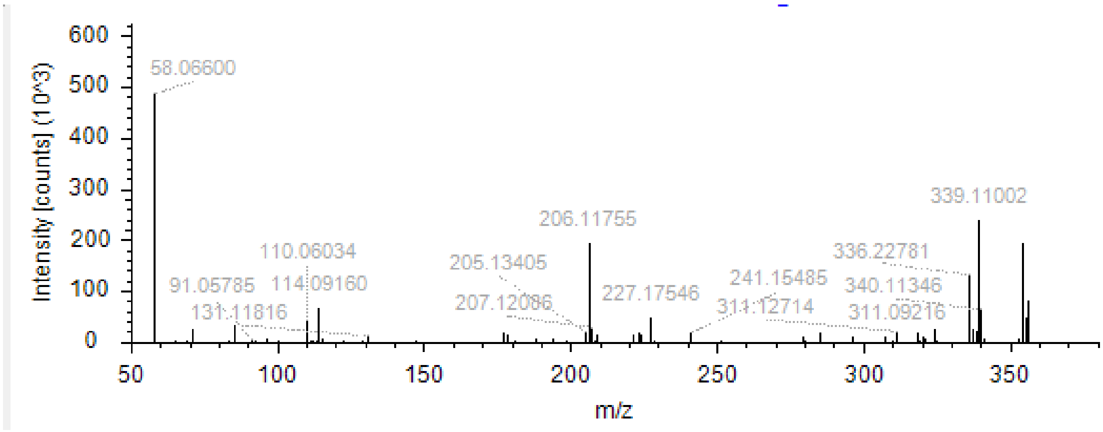

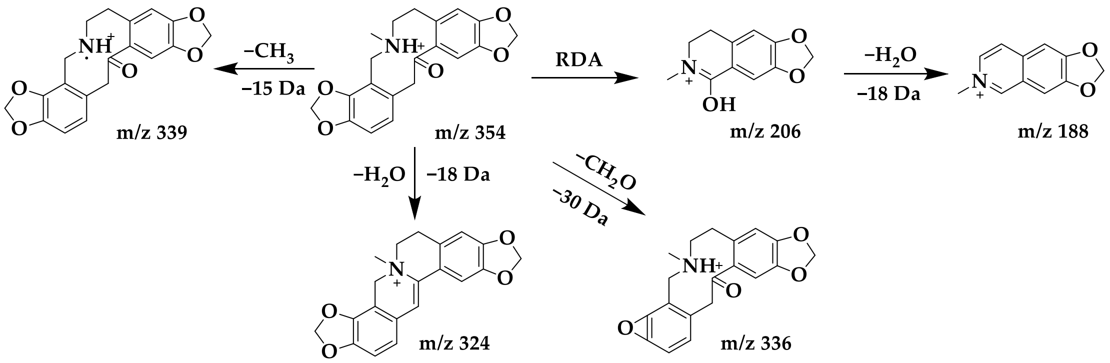

Compounds 10: (S)- Bulbocapnine

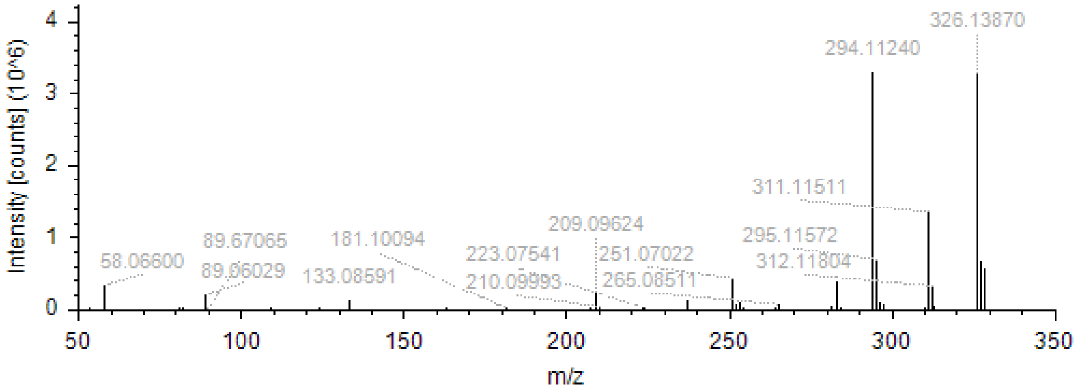

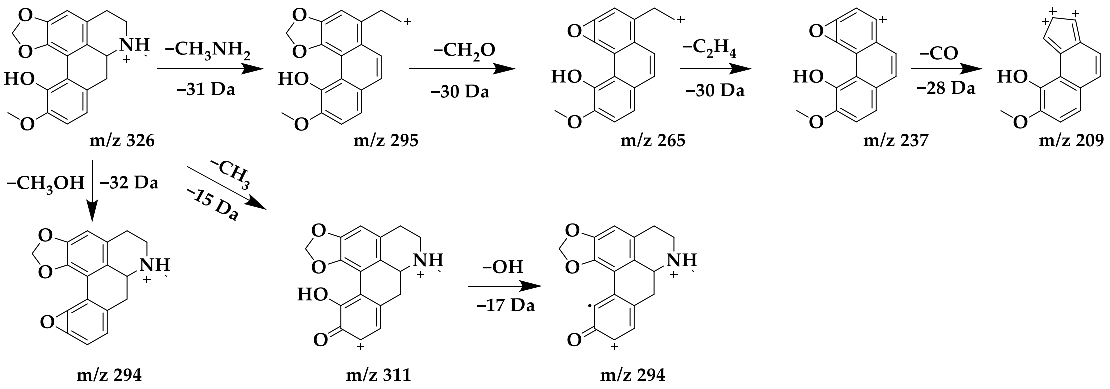

Compounds 11: Apomorphine

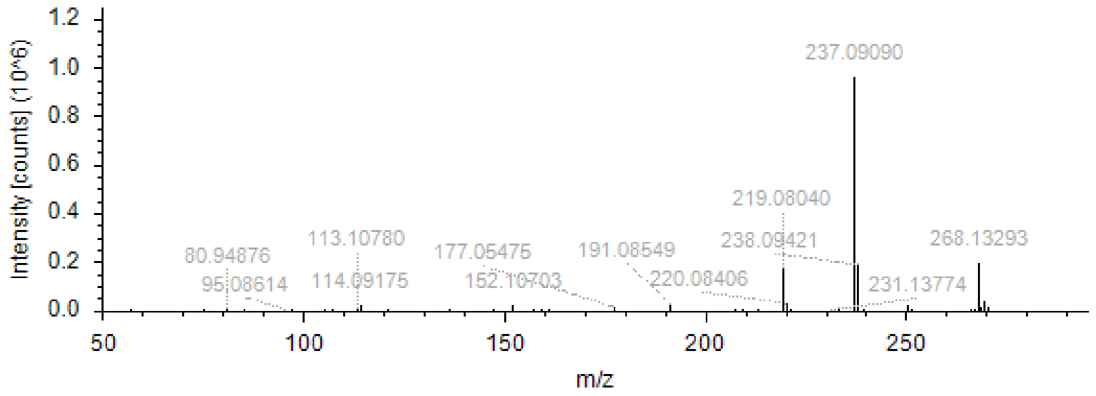

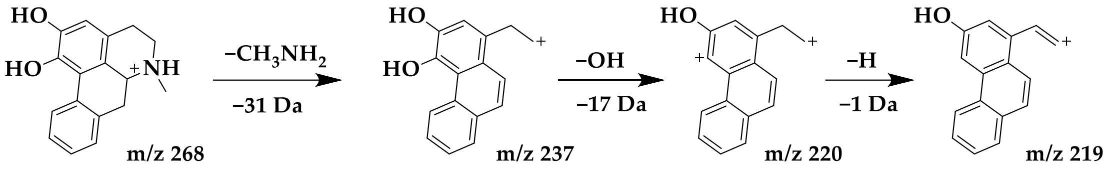

Compounds 12: Norarmepavine

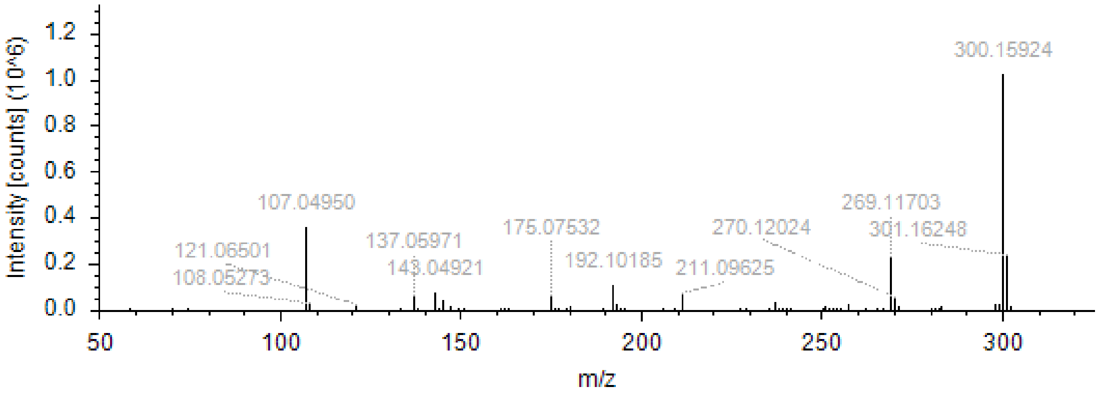

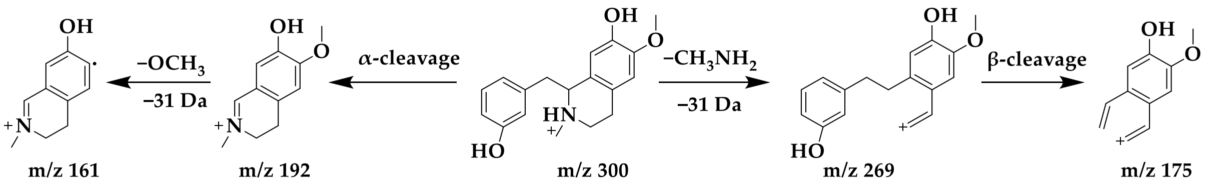

Compounds 13: N-Methylasimilobine

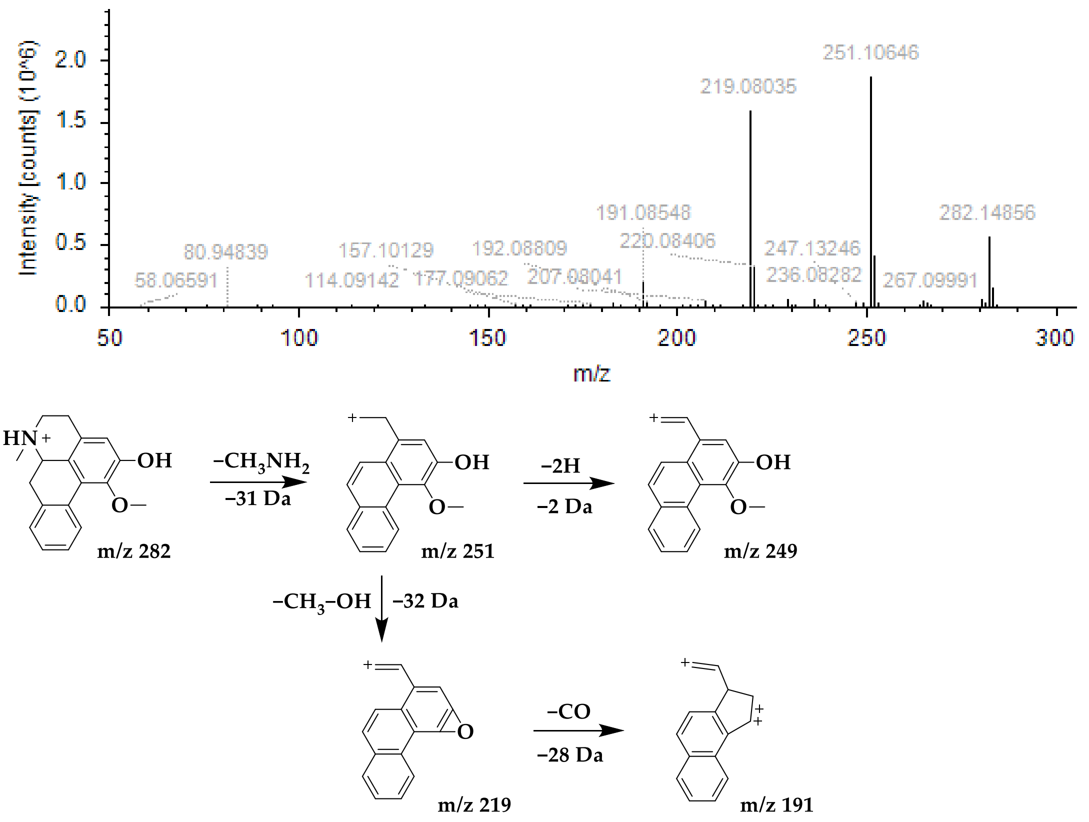

Compounds 14: Isococlaurine

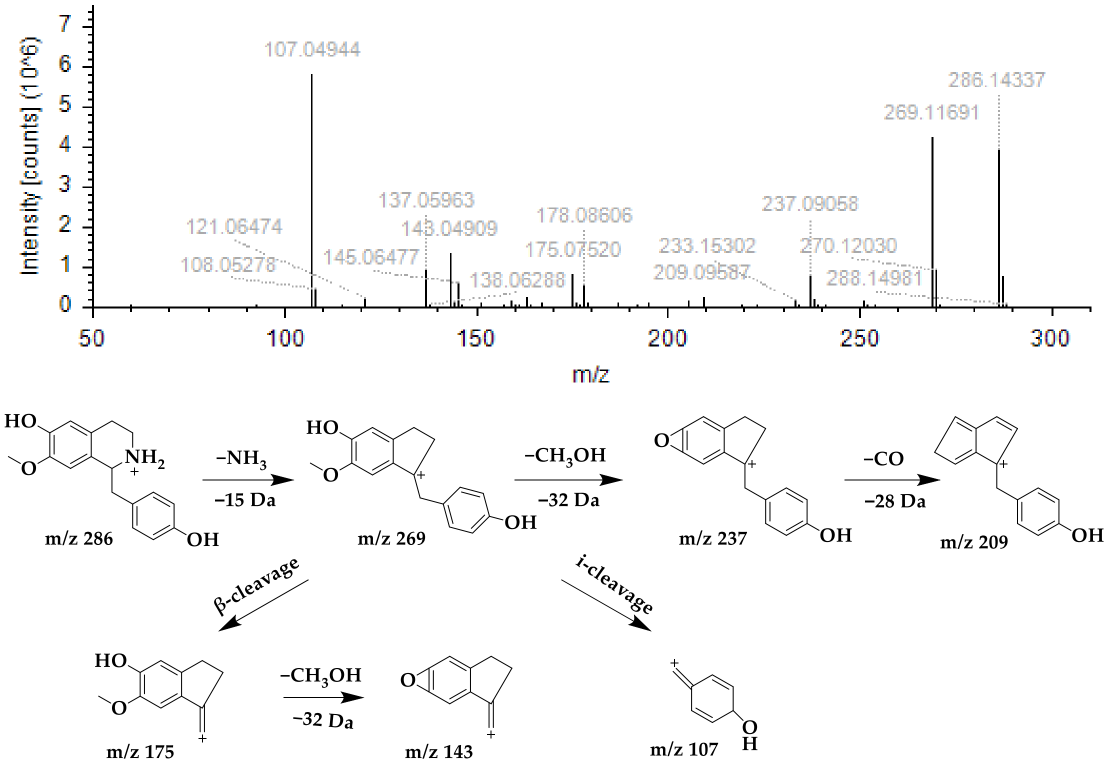

Compounds 15: (S)-Coclaurine

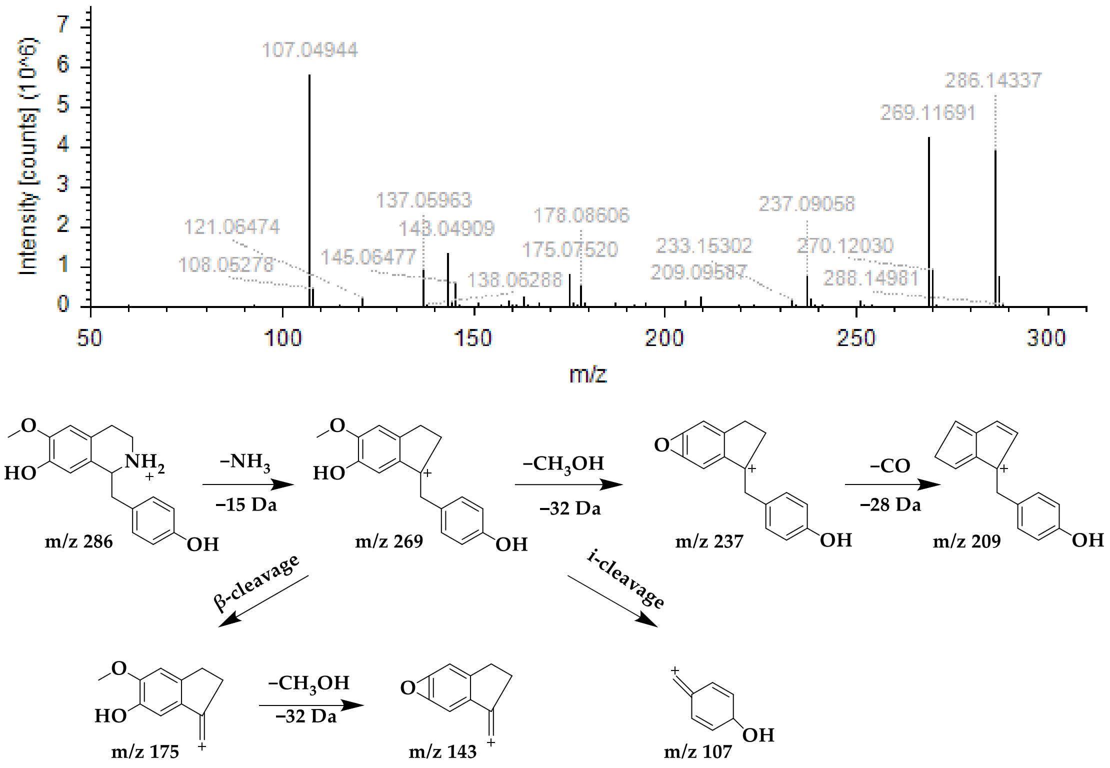

Compounds 16: Asimilobine

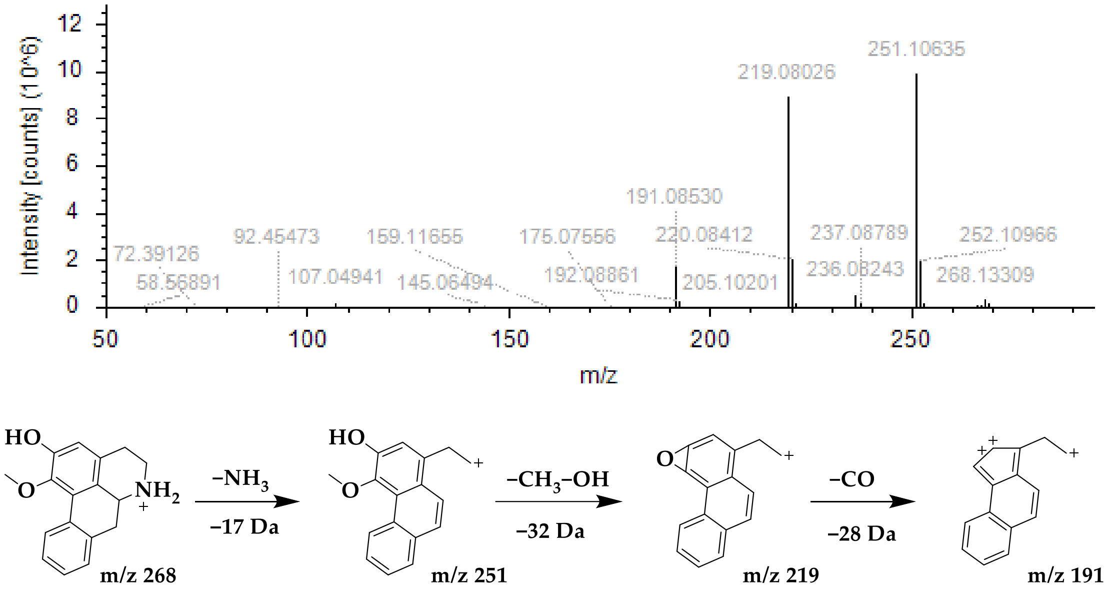

Compounds 17: Ushinsunine

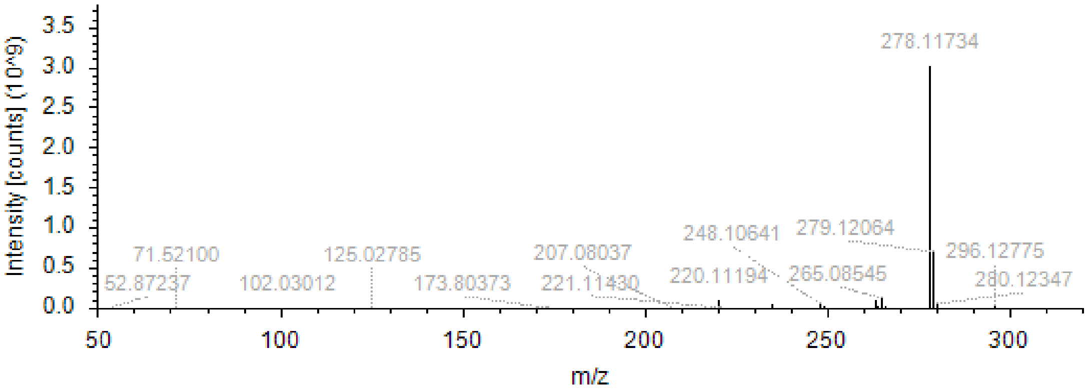

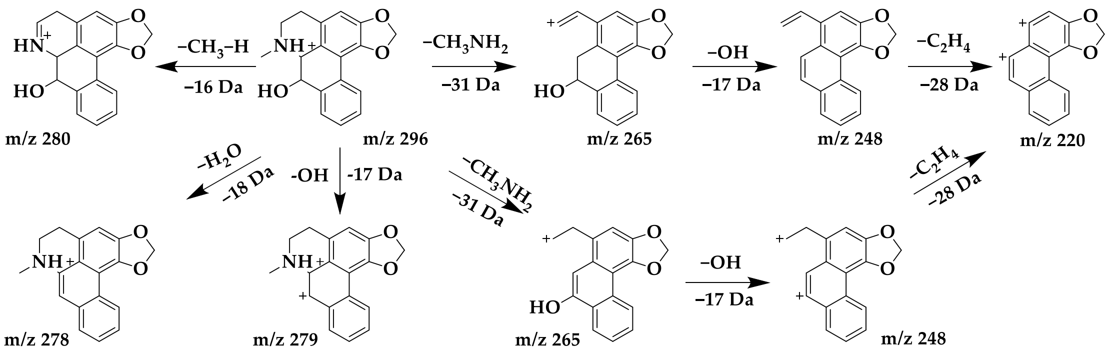

Compounds 18: O-Methylarmepavine

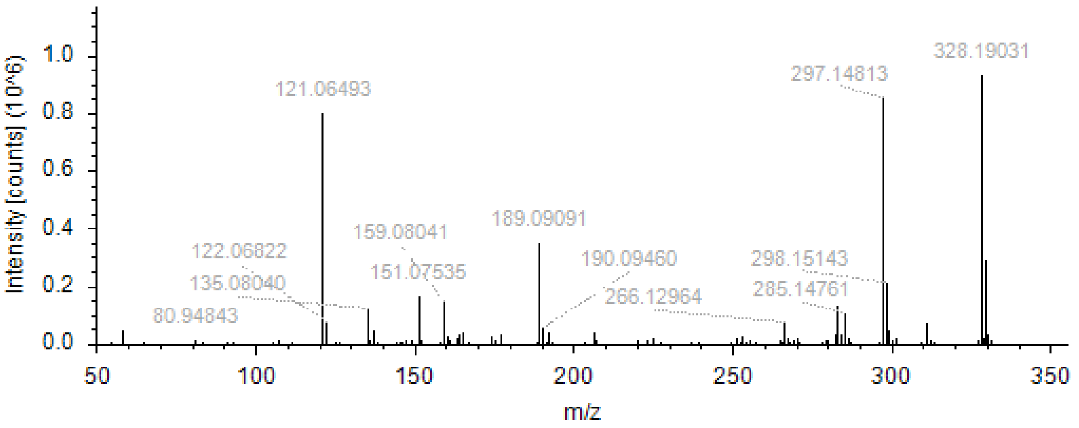

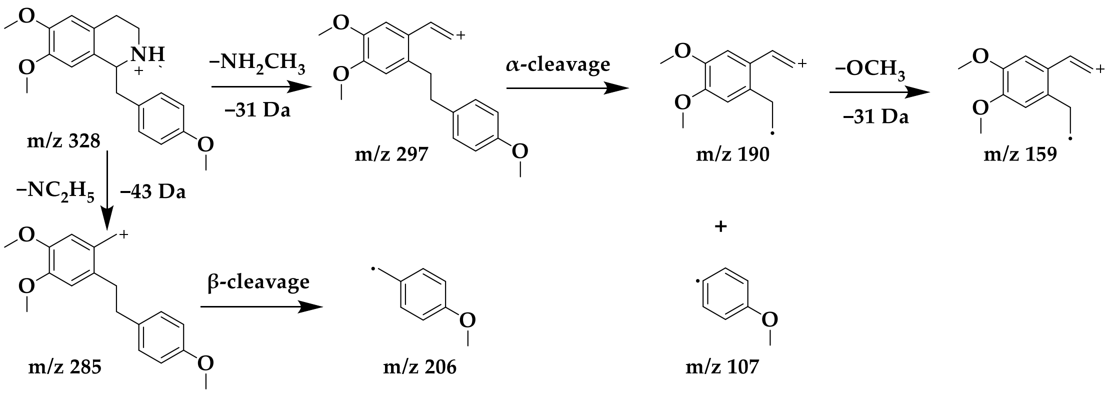

Compounds 19: Corydaline

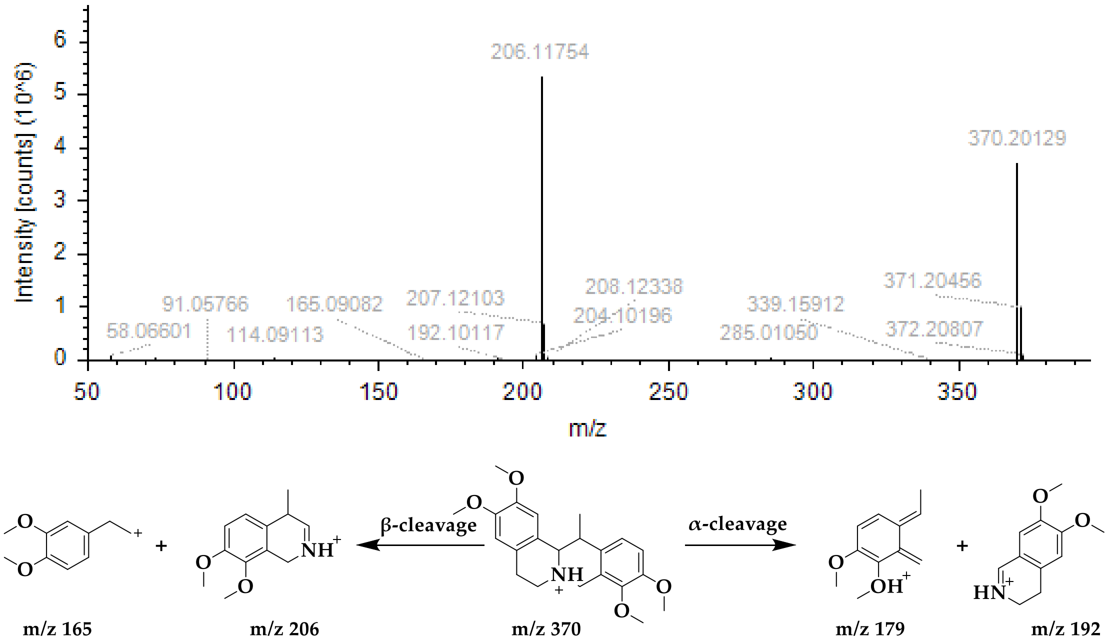

Compounds 20: Menisperine

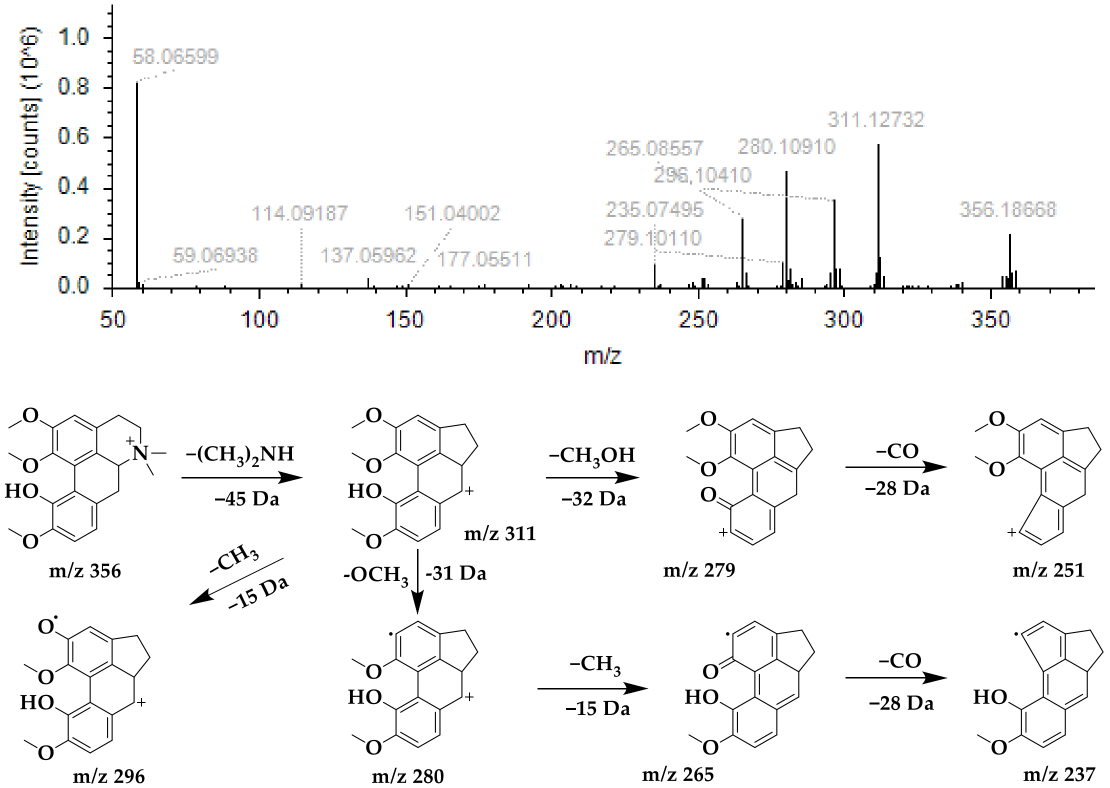

Compounds 21: Palmatine

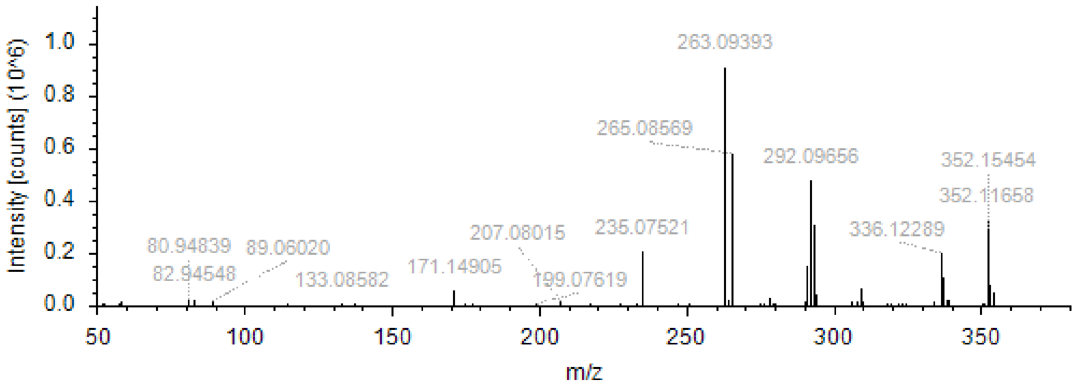

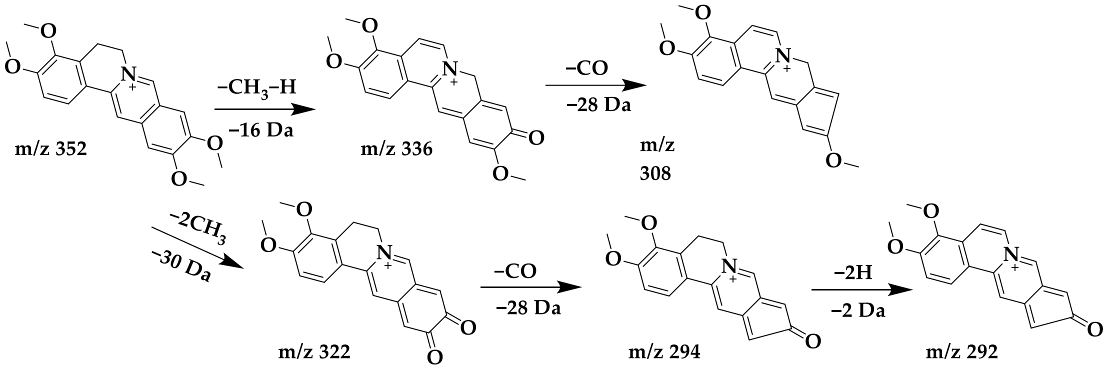

Compounds 22: Chelidonine

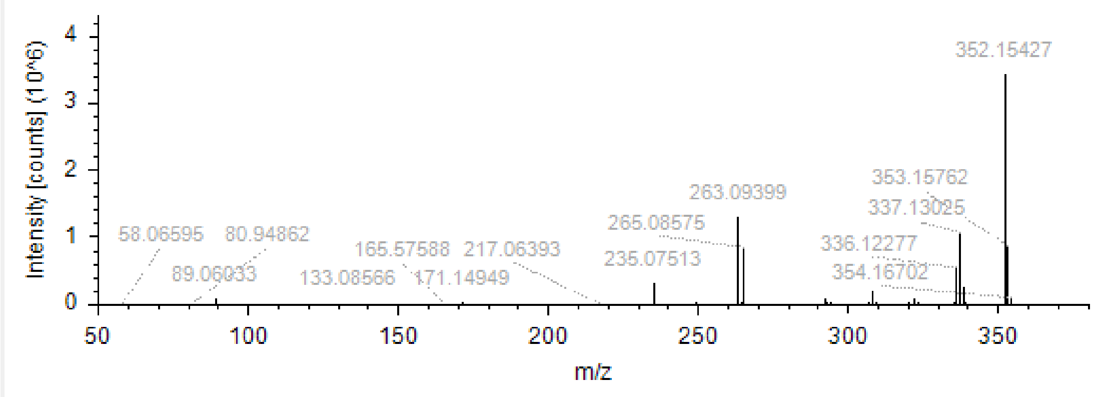

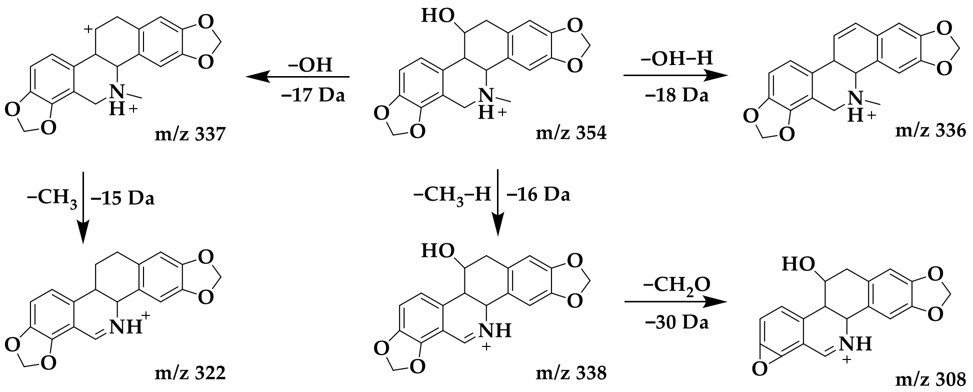

Compounds 23: 8-Methyldihydrochelerythrine

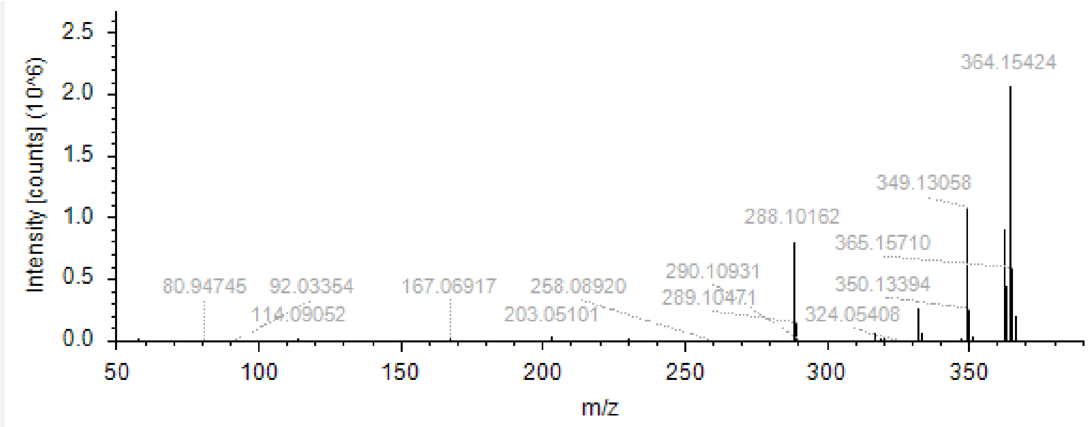

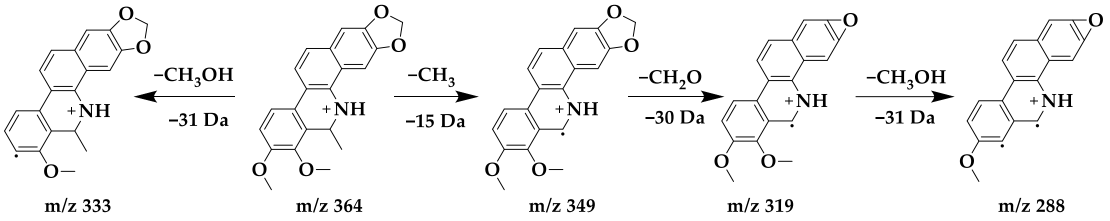

Compounds 24: Anolobine

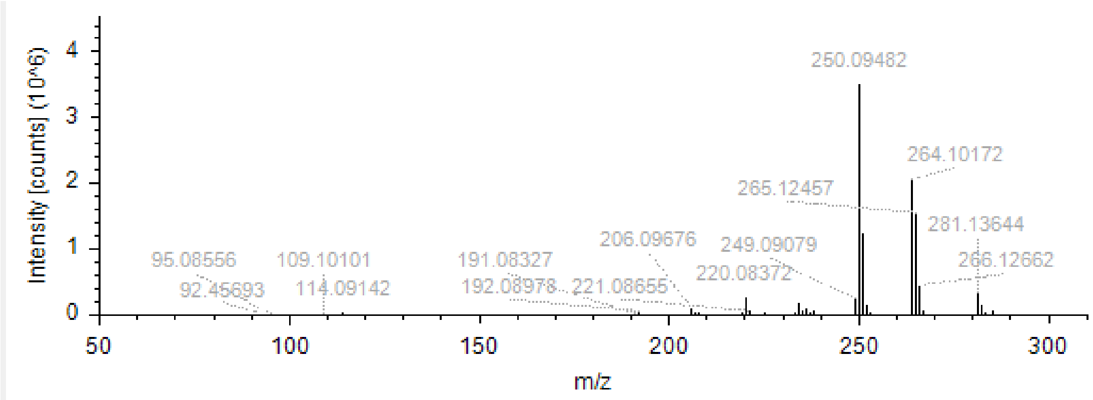

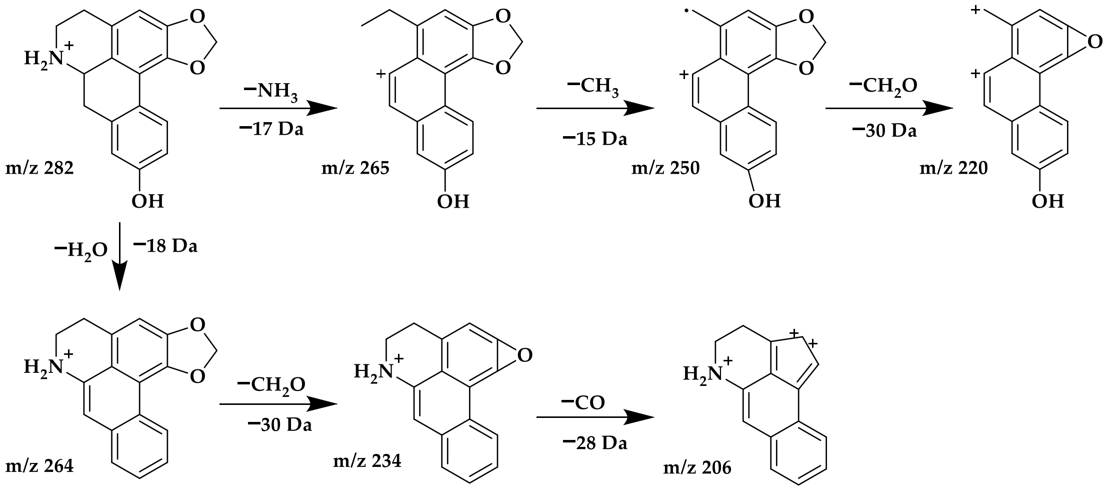

Compounds 25: cheilanthifoline

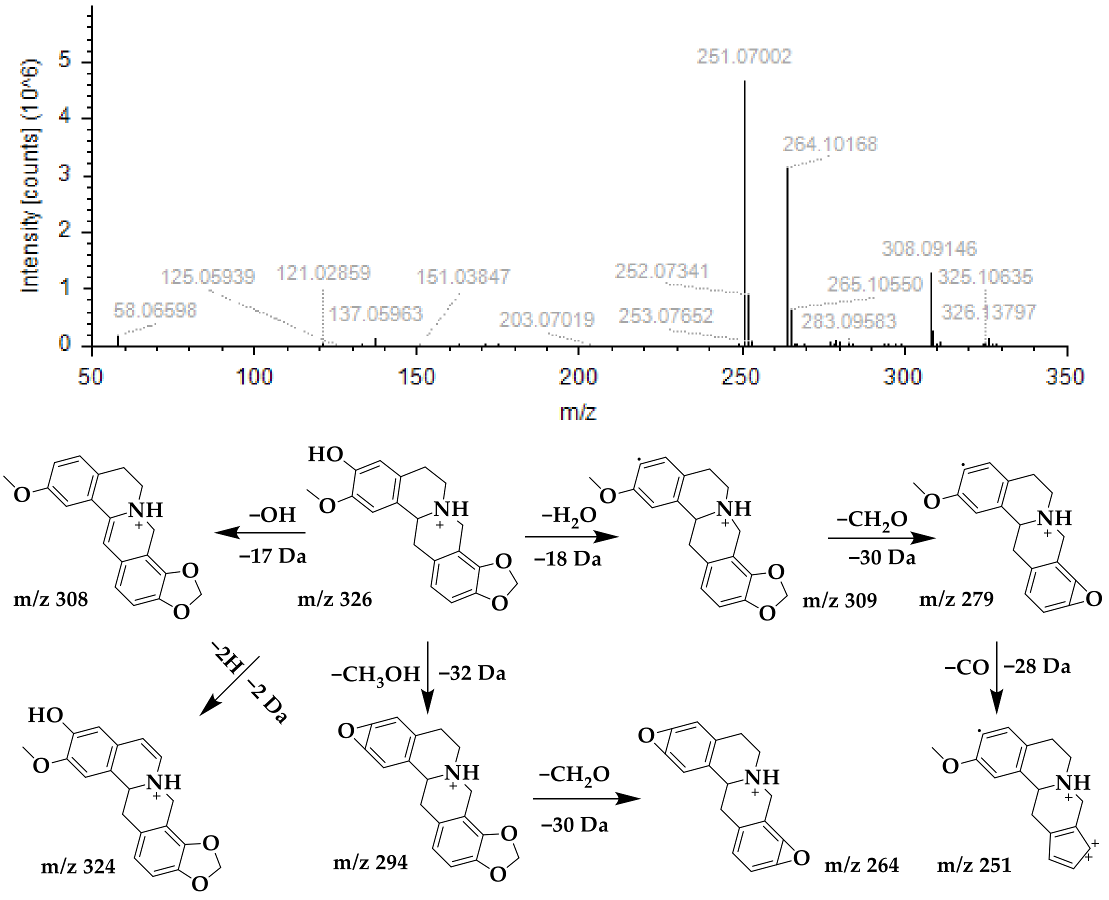

Compounds 26: Chelerythrine

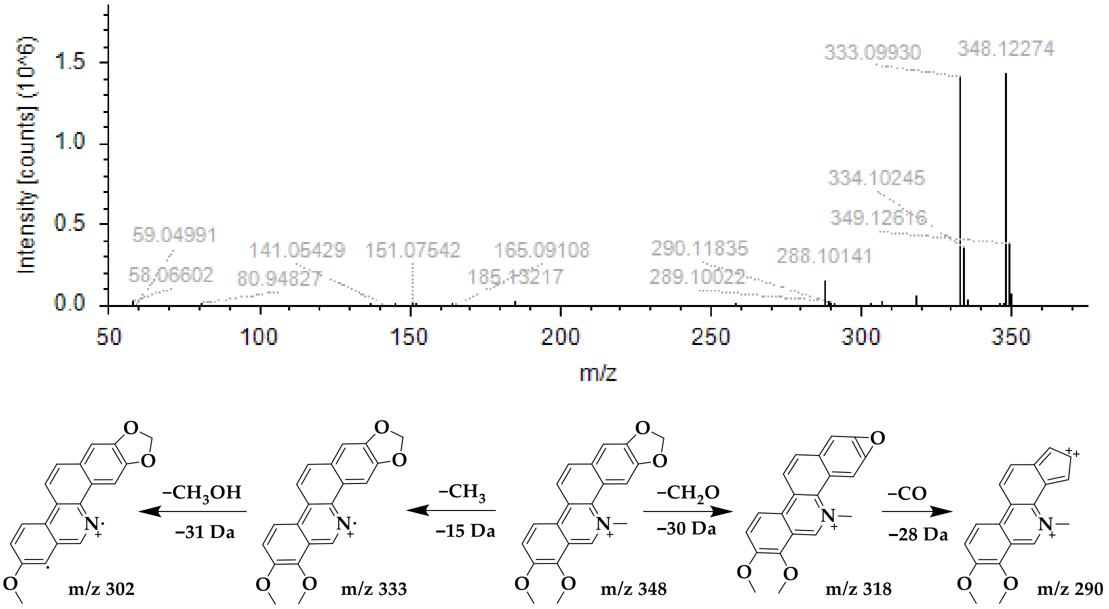

Compounds 27: Roemerine

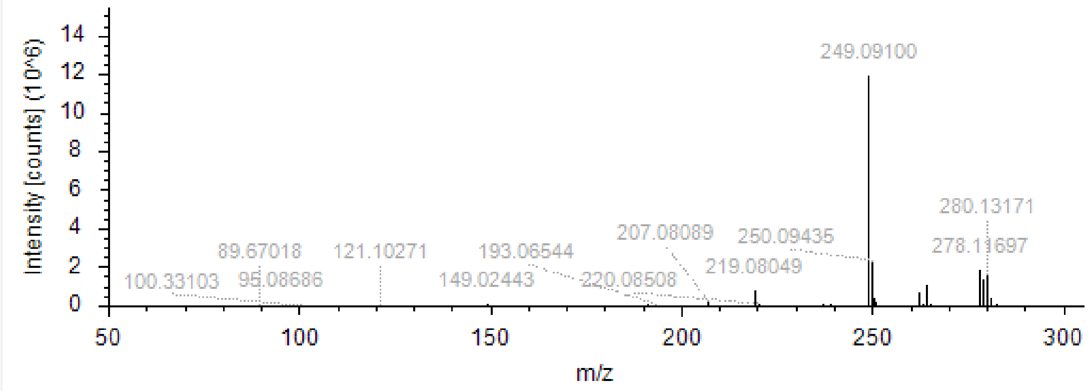

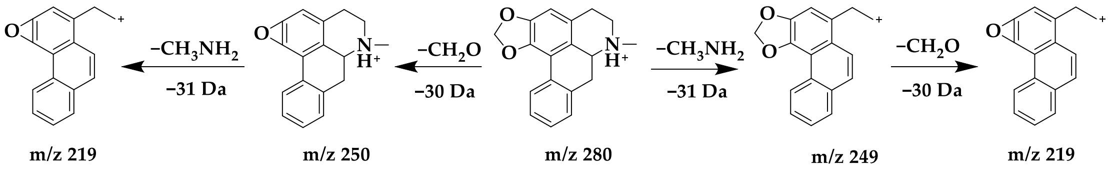

Compounds 31: Xanthoplanine

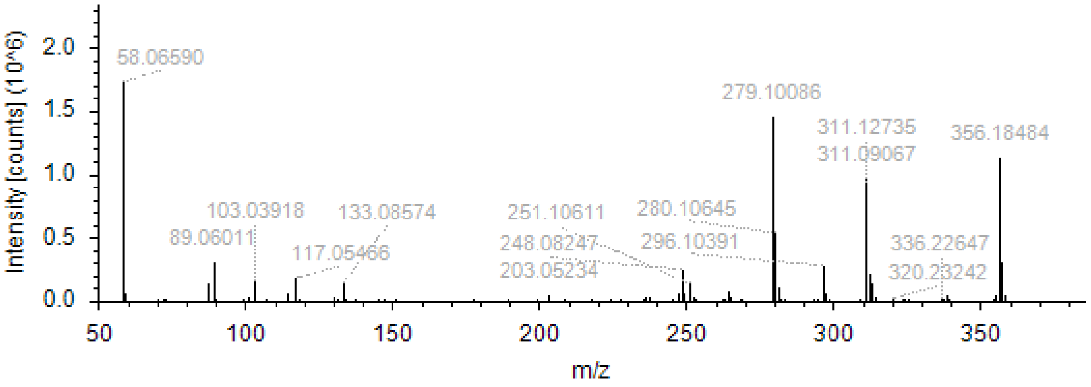

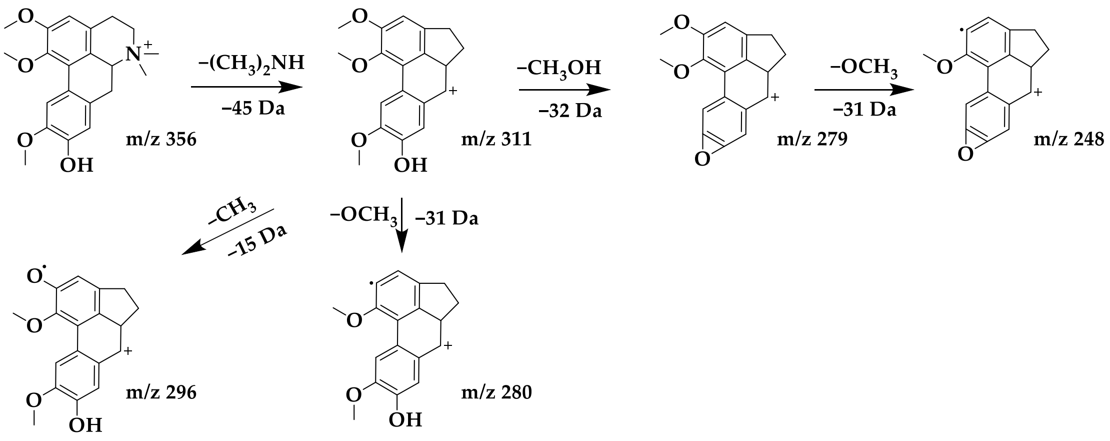

Compounds 32: Armepavine

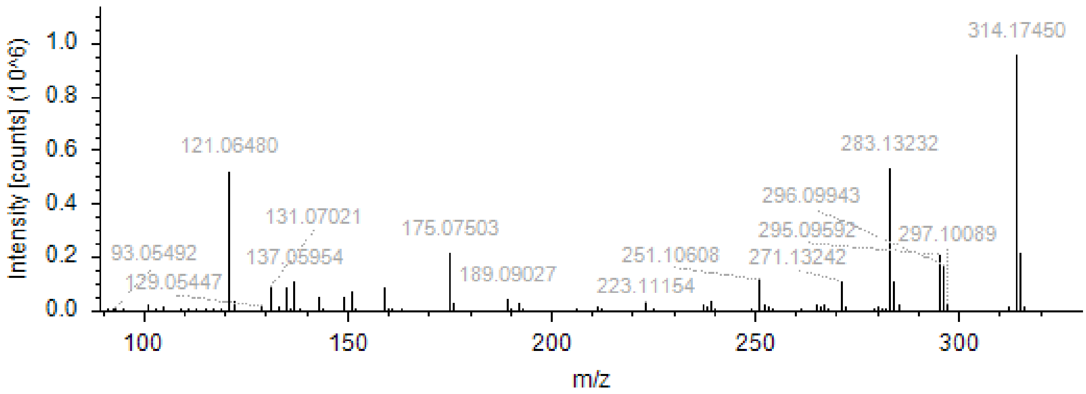

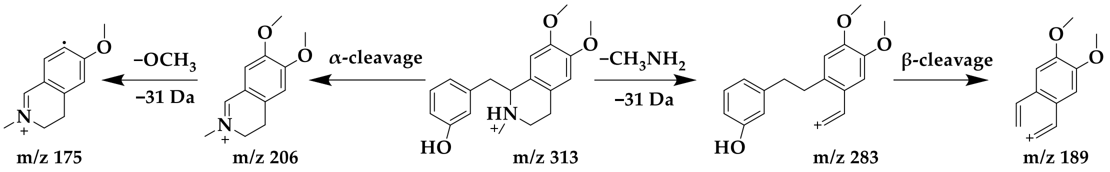

Compounds 33: Michelalbine

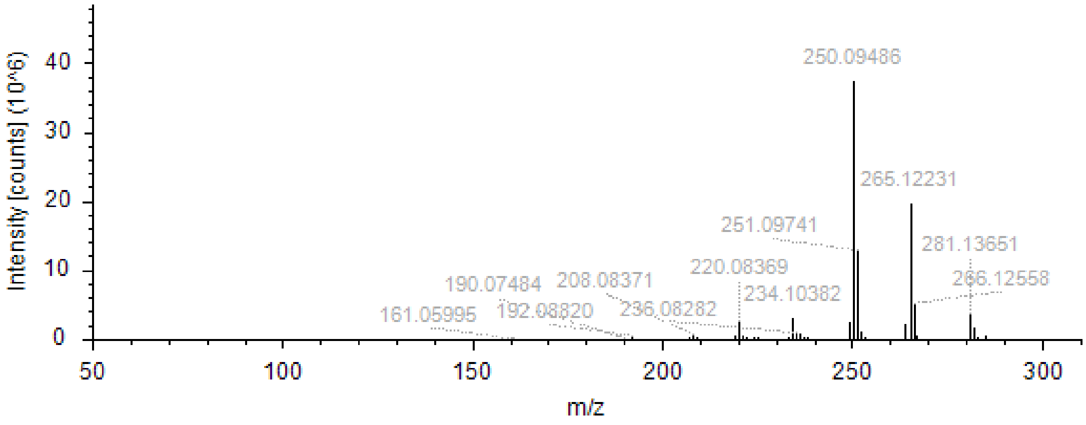

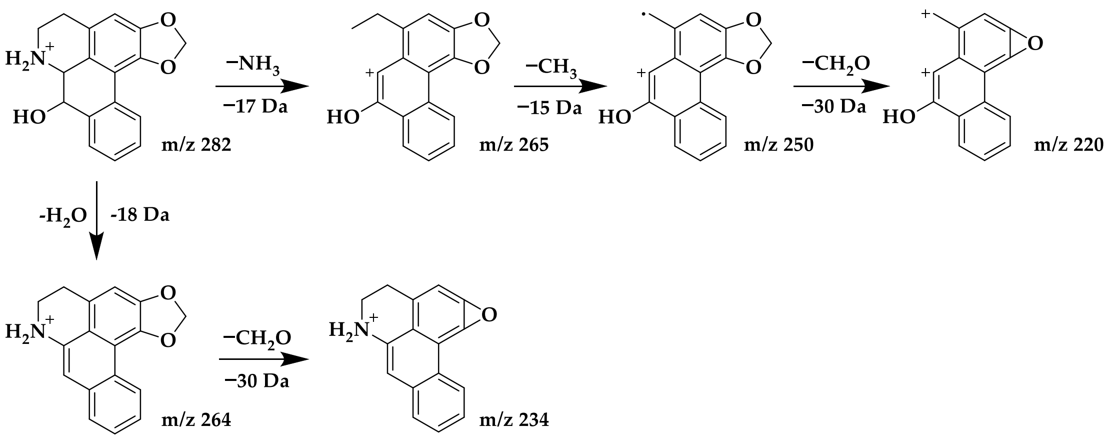

Compounds 34: Tetrahydrocolumbamine

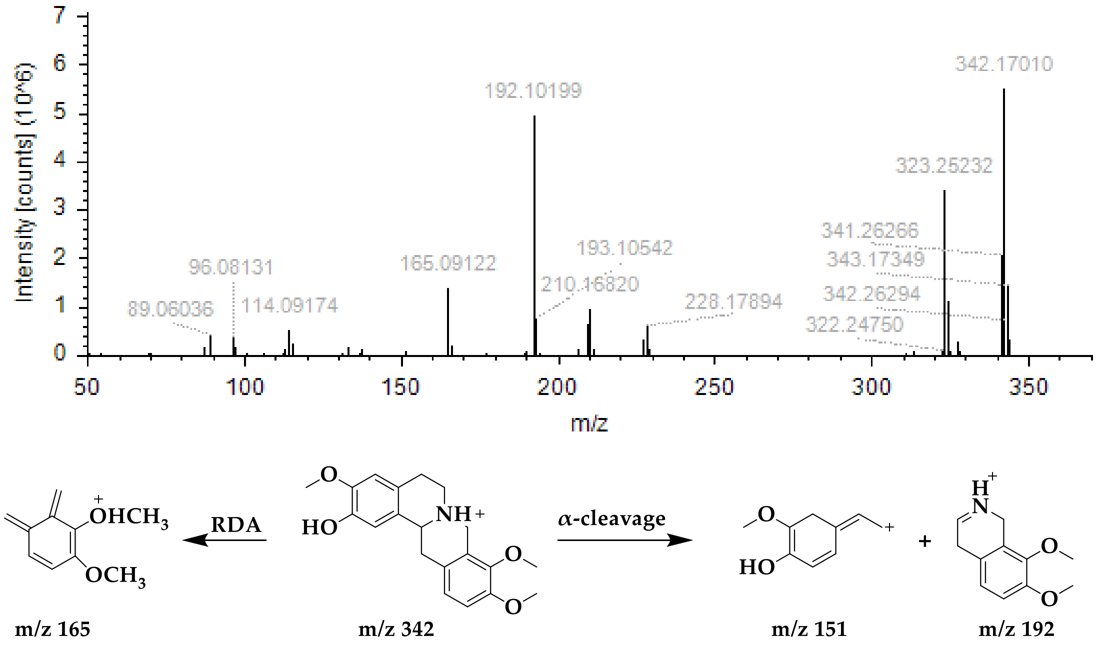

Compounds 37: Magnocurarine

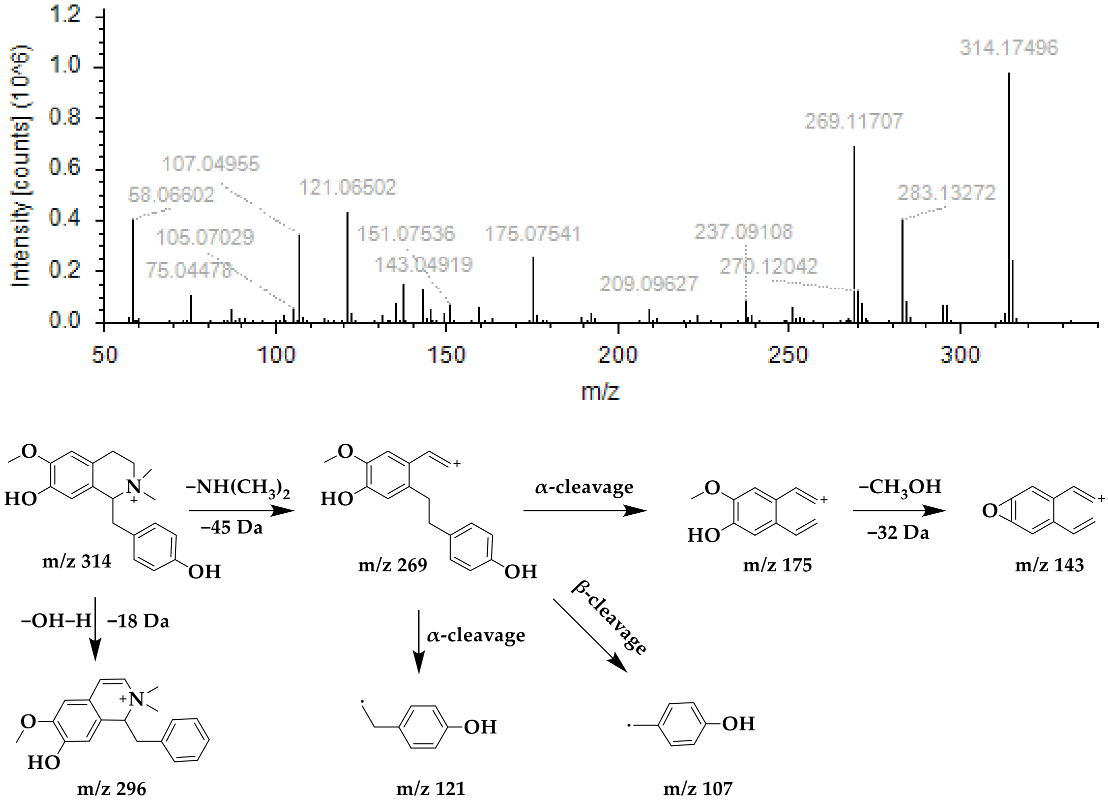

Compounds 38: Allocrytopine

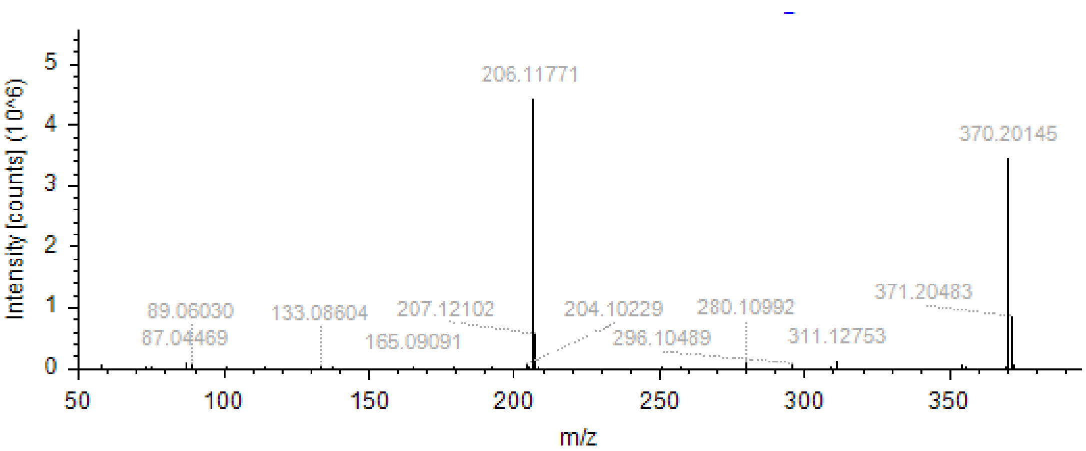

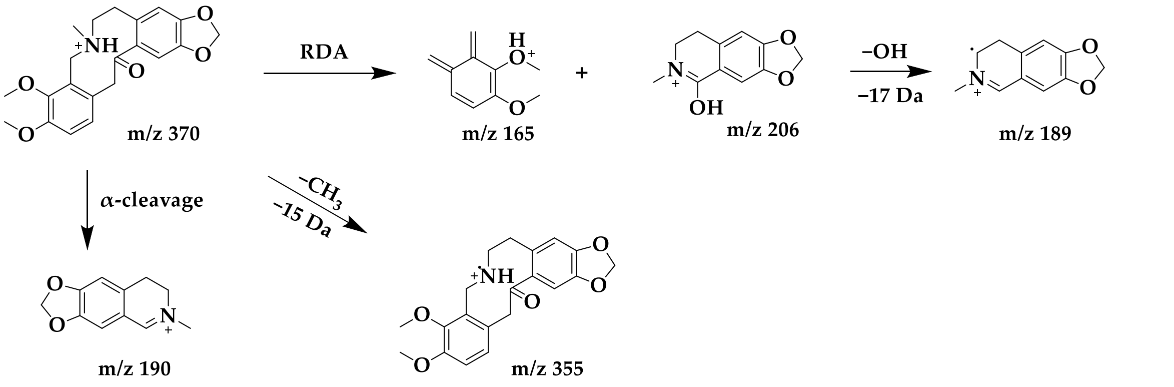

Compounds 39: Laudanosine

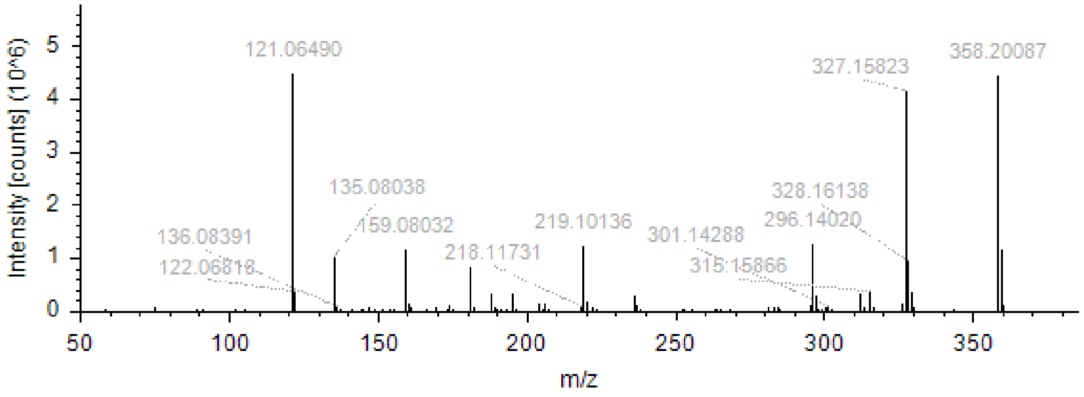

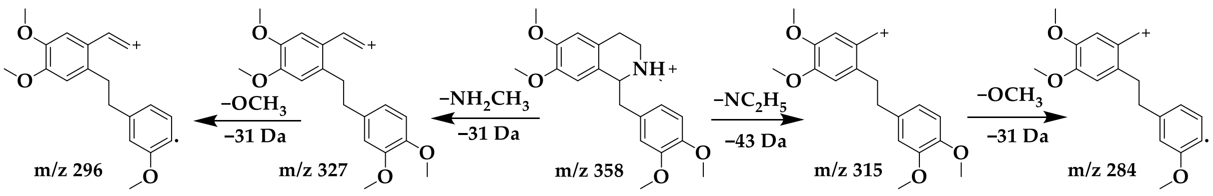

Compounds 41: Anonaine

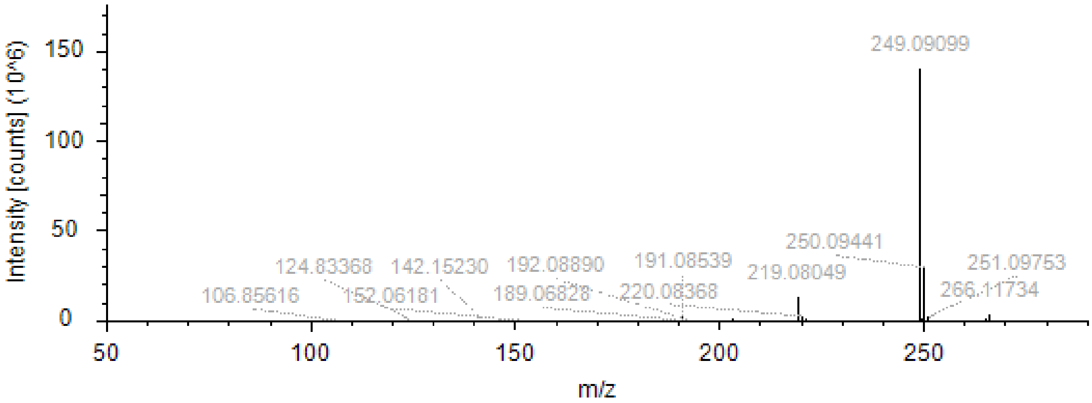

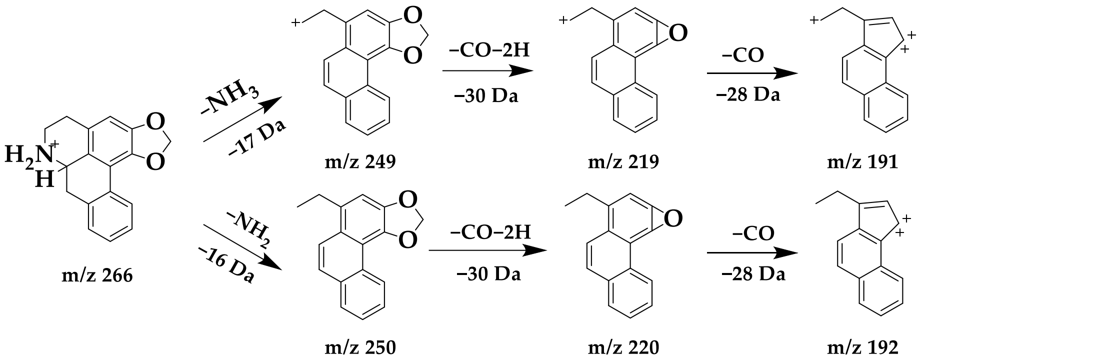

Compounds 42: (S)-Tetrahydropalmatine

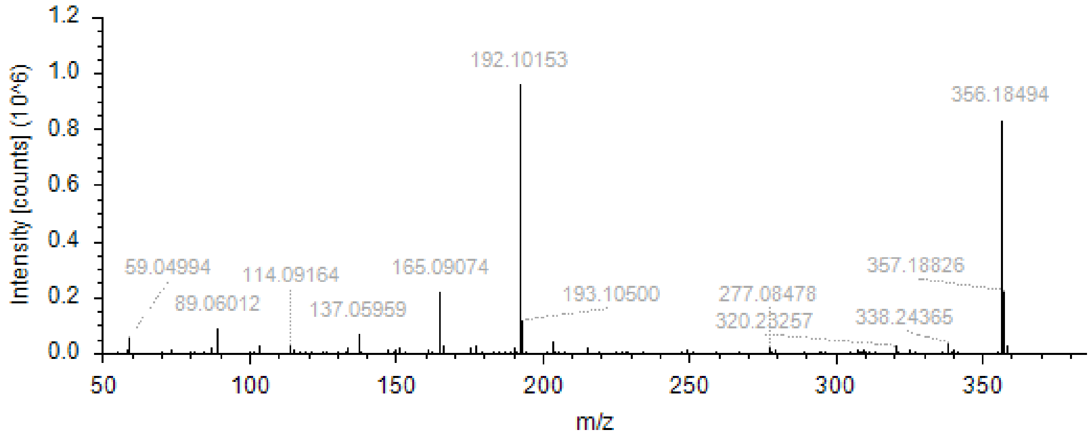

Compound 42: (S)-Tetrahydropalmatine

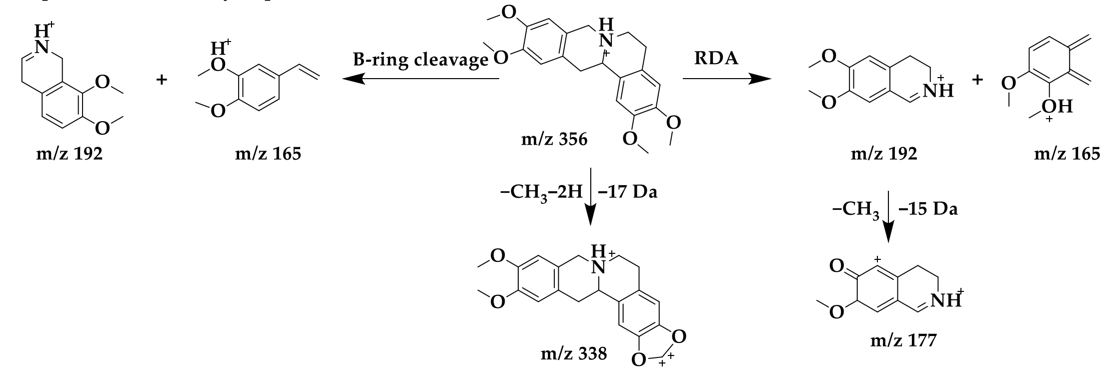

Compounds 43: Protosinomenine

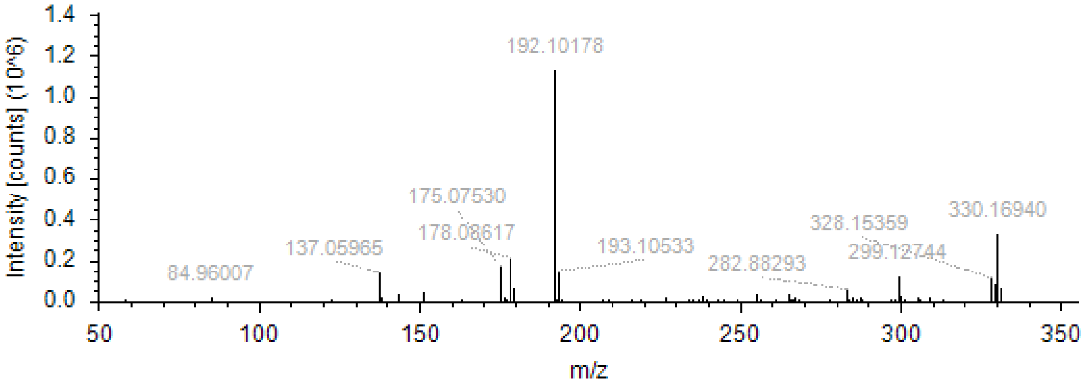

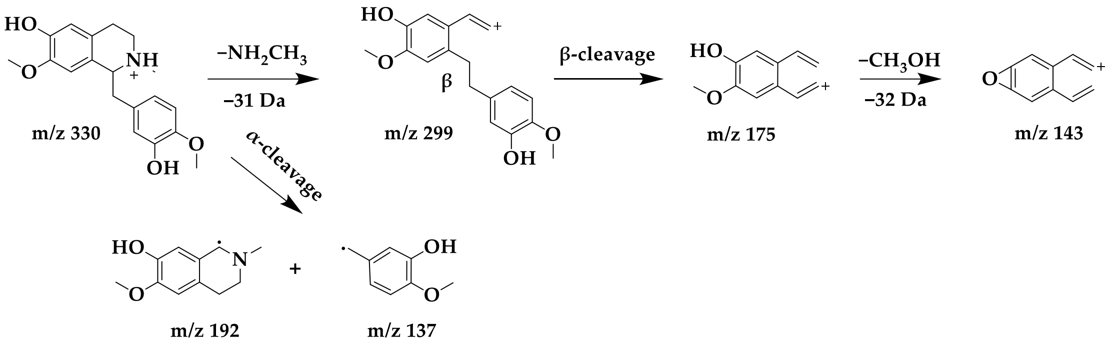

Supplement: Supplementary file 1 [file ijms-24-07972-s001.zip › Supplementary Figure S1.pdf]
