# Supplementary material for: A Study of the Chemical Composition and Biological Activity of Michelia macclurei Dandy Heartwood: New Sources of Natural Antioxidants, Enzyme Inhibitors and Bacterial Inhibitors
Source: Int J Mol Sci. 2023 Apr 28;24(9):7972. doi: 10.3390/ijms24097972 (PMC10177984; doi:10.3390/ijms24097972)
Supplement: Supplementary file 1 [file ijms-24-07972-s001.zip › Supplementary Table S1.pdf]

Supplementary Table S1. The volatile components of MDHW obtained by the two extraction methods.

NO	Compounds	RIcal	RIlit	RA (%)		Type	Identification
				SD	UE		
1	Hexane, 2,3,5-trimethyl-	816	815	-	0.74	FC	MS; RI
2	α-Pinene	935	936	0.20	-	MH	MS; RI
3	Camphene	957	953	0.51	-	MH	MS; RI
4	δ-3-Carene	1013	1011	0.06	0.07	MH	MS; RI
5	Undecane, 4,7-dimethyl-	1213	1212	-	0.14	FC	MS; RI
6	Undecane, 3,8-dimethyl-	1222	1228	-	0.17	FC	MS; RI
7	Dodecane, 2,6,11-trimethyl-	1275	1275	-	**1.44**	FC	MS; RI
8	Decane, 2,3,5,8-tetramethyl-	1321	1318	-	0.69	FC	MS; RI
9	Dodecane, 4,6-dimethyl-	1331	1325	-	0.25	FC	MS; RI
10	Copaene	1367	1377	0.03	0.51	SH	MS; RI
11	Tridecane, 2-methyl-	1372	1360	-	0.10	FC	MS; RI
12	Dodecane, 2,6,10-trimethyl-	1375	1378	-	0.22	SH	MS; RI
13	Cedrene	1388	1380	0.05	-	SH	MS; RI
14	β-Funebrene	1391	1392	0.02	-	SH	MS; RI
15	β-Longipinene	1395	1394	0.02	0.19	SH	MS; RI
16	β-Elemene	1405	1400	**11.88**	**8.20**	SH	MS; RI
17	Sesquithujene	1414	1417	0.42	0.35	SH	MS; RI
18	α-Cedrene	1419	1413	0.04	-	SH	MS; RI
19	α-Bergamotene	1422	1422	0.17	0.12	SH	MS; RI
20	γ-Elemene	1426	1434	0.03	-	SH	MS; RI
21	Caryophyllene	1428	1425	**7.43**	-	SH	MS; RI
22	Isocaryophyllene	1434	1425	0.06	-	SH	MS; RI
23	β-Cedrene	1437	1428	0.03	-	SH	MS; RI
24	1,9-Aristoladiene	1441	1435	0.23	0.15	SH	MS; RI
25	Calarene	1444	1449	0.62	-	SH	MS; RI
26	α-Maaliene	1447	1442	0.22	-	SH	MS; RI
27	β-Vetispirene	1455	-	0.20	-	SH	MS
28	2,6,10-Trimethyltridecane	1459	1461	-	0.33	FC	MS; RI
29	Alloaromadendrene	1466	1462	0.30	0.18	SH	MS; RI
30	α-Himachalene	1469	1451	0.15	-	SH	MS; RI
31	γ-Muurolene	1474	1472	**2.93**	-	SH	MS; RI
32	Acoradiene	1481	1475	0.45	-	SH	MS; RI
33	Isocadinene	1484	1481	0.22	-	SH	MS; RI
34	δ-Bisabolene	1488	1479	1.26	**2.18**	SH	MS; RI
35	α-Curcumene	1492	1488	1.73	-	SH	MS; RI
36	β-Guaiene	1500	1491	0.54	-	SH	MS; RI
37	cis-α-Bisabolene	1502	1504	0.34	-	SH	MS; RI
38	γ-Amorphene	1507	1508	0.15	-	SH	MS; RI
39	α-Muurolene	1515	1507	1.06	-	SH	MS; RI
40	β-Bisabolene	1519	1519	**4.25**	-	SH	MS; RI

41	2,4-Di-tert-Butylphenol	1521	1521		0.60	AC	MS; RI
42	δ-Cadinene	1523	1522	0.37		SH	MS; RI
43	Cadina-1(10),4-diene	1528	1525	0.44	0.44	SH	MS; RI
44	Eudesma-3,7(11)-diene	1533	1537	2.55	-	SH	MS; RI
45	Tetradecane, 2,6,10-trimethyl-	1539	1540	-	0.04	FC	MS; RI
46	Elemol	1543	1546	2.79	0.56	SO	MS; RI
47	Elemicin	1554	1554	0.24	-	AC	MS; RI
48	β-Calacorene	1561	1563	0.48	-	SH	MS; RI
49	trans-Nerolidol	1569	1566	0.13	-	SO	MS; RI
50	Caryophyllene oxide	1574	1570	0.10	-	SO	MS; RI
51	Maalialcohol	1578	1577	1.06	-	SO	MS; RI
52	Gleenol	1586	1587	0.02	-	SO	MS; RI
53	Globulol	1593	1591	0.13	-	SO	MS; RI
54	Spathulenol	1597	1587	0.61	-	SO	MS; RI
55	Guaiol	1600	1600	**10.67**	0.54	SO	MS; RI
56	Viridiflorol	1608	1608	1.47	-	SO	MS; RI
57	β-Eudesmol	1628	1628	**9.68**	**-**	SO	MS; RI
58	Cubenol	1631	1630	0.20	-	SO	MS; RI
59	Epiglobulol	1635	1629	0.43	-	SO	MS; RI
60	Pentadecane, 2,6,10-trimethyl-	1646	1649	-	0.70	SH	MS; RI
61	Epicubenol	1649	1642	**3.92**	-	SO	MS; RI
62	β-Acorenol	1658	1648	1.96	-	SO	MS; RI
63	Cedrelanol	1663	1665	0.47	-	SO	MS; RI
64	Hexadecane, 4-methyl-	1664	1669	-	0.39	FC	MS; RI
65	δ-Cadinol	1669	1670	**4.35**	-	SO	MS; RI
66	Eudesma-4(15),7-dien-1-β-ol	1682	1684	1.36	-	SO	MS; RI
67	β-Bisabolol	1690	1675	**7.14**	-	SO	MS; RI
68	4(15),5,10(14)-Germacratrien-1-ol	1707	1694	0.72	-	SO	MS; RI
69	Farnesol	1713	1710	**9.98**	-	SO	MS; RI
70	6-epi-Shyobunol	1725	1721	0.48	-	SO	MS; RI
71	Crocetane	1805	1813	-	**1.16**	FC	MS; RI
72	Octadecane, 6-methyl-	1841	1842	-	0.43	FC	MS; RI
73	Octadecane, 2-methyl-	1858	1860	-	0.57	FC	MS; RI
74	Heptadecane, 2,6,10,14-tetramethyl-	1867	1867	-	0.18	FC	MS; RI
75	Heptadecane, 2,6,10,15-tetramethyl-	1908	1914	-	**2.23**	FC	MS; RI
76	7,9-Di-tert-butyl-1-oxaspiro(4,5)deca-6,9-diene-2,8-dione	1921	1916	-	**0.84**	AC	MS; RI
77	Metilox	1940	1943	-	0.69	FC	MS; RI
78	Isoeicosane	1952	1962	-	**1.55**	FC	MS; RI
79	Eicosane, 2-methyl-	2055	2062	-	0.10	FC	MS; RI
80	Octadecanoic acid, 2-propenyl	2252	2251	-	0.38	AC	MS; RI

	ester						
81	Methylenebis	2500	-	0.12	-	AC	MS
82	Octadecane, 3-ethyl-5-(2-ethylbutyl)-	2989	-	-	**1.03**	FC	MS
83	Tris(2,4-di-tert-butylphenyl) phosphite	3401	3396	-	**29.26**	FC	MS; RI
84	Tris(2,4-di-tert-butylphenyl) phosphate	3580	3582	-	**18.43**	FC	MS; RI
	Monoterpene hydrocarbons (MH)			0.78	0.07		
	Sesquiterpene hydrocarbons (SH)			38.74	13.23		
	Oxygenated sesquiterpenes (SO)			57.66	0.54		
	Fatty compounds (FC)			0.00	59.93		
	Aromatic compounds (AC)			0.37	2.38		
	Total identified			97.54	76.15		

Note: "-" Indicates that the information is not queried or detected; Bold indicates the top ten compounds; RIcal—Retention Index, calculated by authors; RIlit—Retention Index by literature data; RA: Relative Area; MS: MS/MS spectrum; RI: Retention Index.
