# Supplementary figures and images for: A Study of the Chemical Composition and Biological Activity of Michelia macclurei Dandy Heartwood: New Sources of Natural Antioxidants, Enzyme Inhibitors and Bacterial Inhibitors
Source: Int J Mol Sci. 2023 Apr 28;24(9):7972. doi: 10.3390/ijms24097972 (PMC10177984; doi:10.3390/ijms24097972)

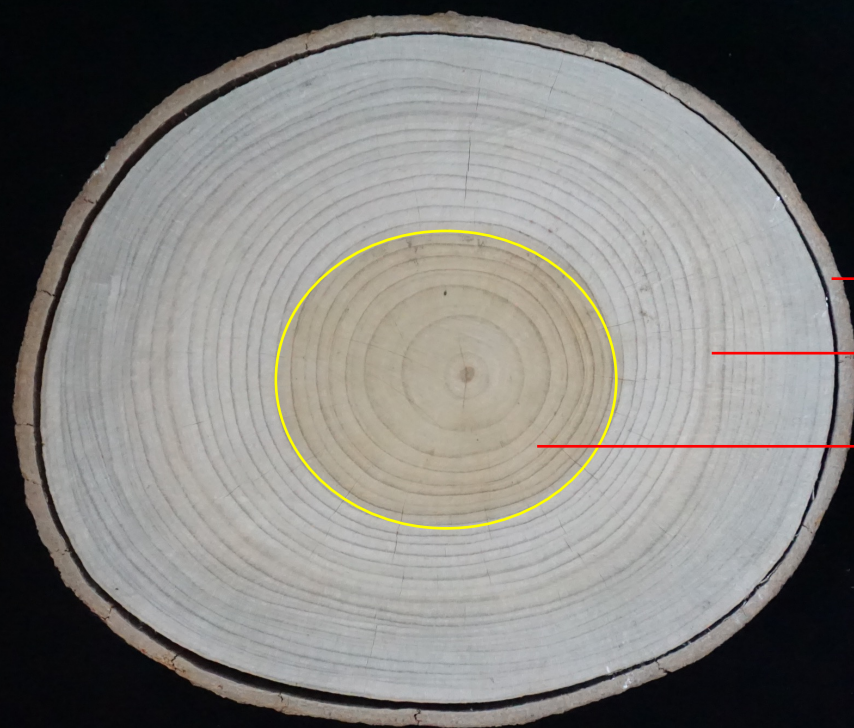

Bark

Sapwood

Heartwood

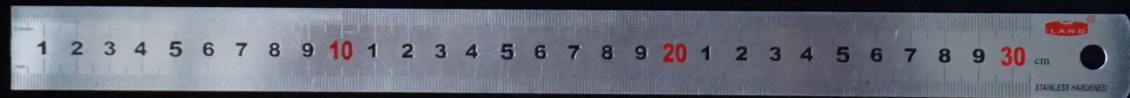

Supplement: Supplementary file 1 [file ijms-24-07972-s001.zip › Supplementary Figure S2.pdf]
